# Does parental and adolescent participation in an e-health lifestyle modification intervention improves weight outcomes?

**DOI:** 10.1186/s12889-017-4220-0

**Published:** 2017-04-24

**Authors:** Andrew W. Tu, Allison W. Watts, Jean-Pierre Chanoine, Constadina Panagiotopoulos, Josie Geller, Rollin Brant, Susan I. Barr, Louise Mâsse

**Affiliations:** 1 School of Population and Public Health, University of British Columbia, 2206 East Mall, Vancouver, BC V6T 1Z3 Canada; 2 School of Public Health, University of Minnesota, 1300 South Second St, Suite 300, Minneapolis, MN 55454 USA; 3 Department of Pediatrics, University of British Columbia, 4480 Oak Street, Vancouver, BC V6H 3V4 Canada; 4 Department of Psychology, University of British Columbia, 2255 Wesbrook Mall, Vancouver, BC V6T 2A1 Canada; 5 Department of Statistics, University of British Columbia, 4480 Oat Street, Vancouver, BC V6H 3V4 Canada; 6 Food Nutrition and Health, University of British Columbia, 2205 East Mall, Vancouver, BC V6T 1Z4 Canada; 7 BC Children’s Hospital Research Institute, School of Population and Public Health, University of British Columbia, 2206 East Mall, Vancouver, BC V6T 1Z3 Canada

**Keywords:** Adherence, e-health, Obesity, Parenting, Lifestyle intervention

## Abstract

**Background:**

Few studies have evaluated the effect of adherence to a lifestyle intervention on adolescent health outcomes. The objective of this study was to determine whether adolescent and parental adherence to components of an e-health intervention resulted in change in adolescent body mass index (BMI) and waist circumference (WC) z-scores in a sample of overweight/obese adolescents.

**Methods:**

In total, 159 overweight/obese adolescents and their parents participated in an 8-month e-health lifestyle intervention. Each week, adolescents and their parents were asked to login to their respective website and to monitor their dietary, physical activity, and sedentary behaviours. We examined participation (percentage of webpages viewed [adolescents]; number of weeks logged in [parents]) and self-monitoring (number of weeks behaviors were tracked) rates. Linear mixed models and multiple regressions were used to examine change in adolescent BMI and WC z-scores and predictors of adolescent participation and self-monitoring, respectively.

**Results:**

Adolescents and parents completed 28% and 23%, respectively, of the online component of the intervention. Higher adolescent participation rate was associated with a decrease in the slope of BMI z-score but not with change in WC z-score. No association was found between self-monitoring rate and change in adolescent BMI or WC z-scores. Parent participation was not found to moderate the relationship between adolescent participation and weight outcomes.

**Conclusions:**

Developing strategies for engaging and promoting supportive interactions between adolescents and parents are needed in the e-health context. Findings demonstrate that improving adolescents’ adherence to e-health lifestyle intervention can effectively alter the weight trajectory of overweight/obese adolescents.

## Background

Childhood obesity continues to be a worldwide epidemic [24, 41] and is associated with significant health issues, including metabolic, cardiovascular, gastrointestinal, pulmonary, orthopedic, and psychological disorders, in adulthood [23]. Among Canadian youth, 31.1% are overweight or obese and 11.6% are obese [31]. Family-based interventions that target physical activity (PA), sedentary, and dietary behaviours have had some success at treating childhood obesity [19, 26]. However, there is a need to improve the efficacy of these interventions among adolescents as they have only demonstrated modest and short-term effects (on average, −0.14 change in body mass index (BMI) z-score at 12 months follow-up) [26].

Web-based or e-health interventions delivered through the internet are a potentially cost-effective and promising method for delivering adolescent weight management interventions as the majority of households (at least 83% of Canadian and U.S. households) have access to the internet [14, 34]. E-health interventions have been shown to be at least as effective as traditional non-web-based interventions [42] and evidence regarding their efficacy to treat or prevent childhood obesity is still emerging [10, 11, 15]. However, adherence (defined as attendance and utilization of intervention components) to e-health interventions varies greatly. In a systematic review of e-health interventions among adults, an average adherence rate of 50% was documented, with a wide range reported across studies (10 to 90%) [18, 34]. Adherence to lifestyle interventions has consistently predicted reduction in BMI z-score in children [13, 36] and recently has been identified as the single most important factor to target for increasing the success of such interventions [27].

E-health interventions often consist of several components (e.g., educational materials, self-monitoring, goal-setting, etc.) and the extent to which adherence to these components affect change in BMI among adolescents is unknown. In one study, adolescents who utilized more self-monitoring components of a lifestyle intervention (i.e., tracking food and beverage intake) achieved greater reductions in BMI z-score [32]. In contrast, other studies of adolescents found no change in BMI z-score with attendance and utilization of program components [28]. Parent participation in interventions can also affect adolescents’ adherence and result in greater weight loss [35, 38, 46]. For instance, when mothers regularly attended treatment sessions with their daughters in a 16-week behavioral weight control program for Black adolescent girls, daughters lost significantly more weight than when mothers did not attend the sessions [38]. Although several e-health weight management interventions for adolescents have involved parental participation, few have evaluated the effect of parental participation on adolescent adherence [1].

In summary, parental and child adherence appears important to improve the efficacy of pediatric weight management interventions. However, different components may have different effects on weight outcomes and the role of parental participation on adolescent adherence has not been well studied in the e-health context. Therefore, this study examined: 1) whether adolescent adherence to specific components of an e-health lifestyle modification intervention (participation and self-monitoring) predicted change in BMI and waist circumference (WC) z-scores and 2) whether parental participation and self-monitoring moderated the relationship between adolescent adherence and change in BMI and WC z-scores. It was hypothesized that adolescents who had higher participation and utilized more self-monitoring tools would have a greater reduction in BMI and WC z-scores compared to adolescents who had lower participation and utilized less self-monitoring tools. In addition, an even greater reduction in BMI and WC z-scores was expected among adolescents with a high participating parent compared with a low participating parent.

## Methods

### Sample

Adolescents and one corresponding parent were recruited through online and paper advertisements (68%), by mailing invitations to patients of the British Columbia (BC) Children’s Hospital Endocrinology and Diabetes Clinic (14%), previous participants of the Centre for Healthy Weights program in BC (14%) and by other sources (4%). Adolescents were eligible if they were between the ages of 11 and 16 years and overweight or obese (defined as >1 Standard Deviation (SD) for age and sex using the World Health Organization (WHO) cut-offs) [44]. The adolescent and their parent had to be living in the Metro Vancouver area, not planning to move during the study and be literate in English. Adolescents were excluded if they were diagnosed with type 1 diabetes, had other comorbidities requiring medical attention, had health problems or disabilities precluding them from being physically active, had a history of a diagnosed psychiatric condition or substance abuse, were enrolled in another behavioural change or weight loss intervention, or were using medications that affected body weight. One parent had to volunteer to participate in the study, the family self-selected which parent was able to attend all in-person assessments. Of the 454 parent-adolescent dyads that responded to the advertisements or invitations and were contacted, 227 declined and 51 did not meet the eligibility criteria. The remaining 176 (38.8%) families were invited to attend and completed the initial baseline assessment −16 dropped out after baseline assessment and were never briefed about the intervention and one family was excluded from the analyses due to a death within the family leaving 159 families included in the analyses.

### Data collection protocol

This study was approved by the University of British Columbia Children’s and Women’s Research Ethics Board and by the University of Waterloo’s Research Ethics Board. Parent-adolescent dyads who met eligibility criteria and expressed interest in the study received the consent form via email and attended an in-person meeting at an evaluation centre. At the initial meeting, the parent-adolescent dyads reviewed and signed the consent forms and completed the baseline questionnaires. One to two weeks later, the dyads returned to the evaluation centre and were introduced to the MySteps® intervention, provided with login information, given pedometers to monitor their steps, and shown how to record and track their behaviours (steps, sedentary behaviours, and dietary intake). Parent-adolescent dyads were instructed to login every week for a period of eight months with new content being available every Sunday. Follow-up assessments were scheduled at 4- and 8-months, and all data were collected from December 2010 to March 2013.

### Intervention

The intervention has been described elsewhere [20]. Briefly, the MySteps® intervention adapted the adolescent PACE e-health intervention [28, 29] to the Canadian context –aligning the intervention with the Canada Food Guide [16] and recommendation for PA [37]. MySteps® included individualized and familial web-based weight management information for adolescents and their parents based on the Chronic Care Model, [39]. Social Cognitive Theory, [5] and the Transtheoretical Model of Change [30]. From these perspectives, the MySteps intervention included motivational counseling via email and telephone contact, skill building techniques (including goal setting, self-monitoring, and social support techniques), tailored interactions, targeted known mediators of behavior change (self-efficacy, barriers, enjoyment, goal setting, and social support), and referral to community resources.

The 8-month (34 weeks) web-based intervention consisted of weekly logins to a website that encouraged healthy eating, PA, and reduced screen time. For the first 17 weeks, adolescents were expected to login on a weekly basis and received new topics, challenges, and skills to help them change their behaviours. The intervention started by assessing each adolescent’s current behaviours and then developing an action plan based on their initial behaviours. During the first 17 weeks, adolescents learn the benefits of improving their health behaviours and set behaviour change goals. In addition, the website allowed them to track their steps, diet, and screen time. For the remaining 17 weeks, adolescents were still expected to login on a weekly basis; however, they were allowed to choose the behaviours and skills they worked on. The parents were asked to login to a different website and each week they received complementary topics and challenges designed to support their child’s challenges of the week. Parents also received bi-weekly emails with information about how to help and encourage their adolescents to change their health behaviours and create a supportive environment. Both parents and adolescents were given a one-week break from logging in to the website, on week 23, but were encouraged to practice what they learned. Weekly reminders to login to the website were emailed to parents and adolescents. In addition, adolescents completed counselling sessions via telephone (5–10 min in duration) on weeks 2, 4, 8, 12, and 16. Finally, both parents and adolescents were provided with pedometers at the start of the intervention to track their behaviors.

### Measures

#### Participation rate

For adolescents, participation rate was defined as the mean percentage of webpages viewed per week, where a total of 83 and 78 pages could be viewed in the first and last 4-months, respectively (typically there were four to five pages per week to view). Parental participation rate was defined as the percentage of weeks the parents logged in to their website over the study period.

#### Self-monitoring

Parent and adolescent self-monitoring rates were defined as the number of weeks parents and adolescents, respectively, tracked either their diet (e.g., consumption of fruit and vegetables, fast foods, and sugar-sweetened beverages), steps, or TV/computer usage divided by the total number of weeks in the study period. The tracking tools were incorporated in the MySteps® and participants had access to the tracking tools as soon as they began the intervention and were prompted throughout the intervention to utilize them. In the first 16 weeks of the intervention they were prompted 11 times to use the internal tracking forms and for the remaining of the program tracking prompts depended on the behavior adolescent chose to work on. Participants needed to have recorded information on at least one of the three activities on at least one of the seven days of the week to be counted as having tracked (yes/no) that particular week.

#### Anthropometric measure

Height, weight, and WC were measured for adolescents and the corresponding parent at baseline, 4-months, and 8-months. Measurements were taken twice at each visit using a stadiometer (Hohltain, United Kingdom) for height, a calibrated scale (Model 597 K, Health-o-meter, McCook, Illinois) for weight, and a measuring tape (Hoechstmass, Germany) for WC. BMI (kg/m^2^) was determined by dividing the average weight (kg) with the average height (m) squared. BMI z-scores were derived from a Stata macro developed by the WHO for children and adolescents aged 5 to 19 (World Health Organization [45]). WHO cutoffs for overweight and obesity were used to describe the weight status of adolescents (>1 standard deviation (SD) = overweight; >2 SD = obesity) and parents (<18.5 as underweight, 18.5 to 24.9 as normal weight, 25 to 29.9 as overweight, and > = 30 as obese). WC z-scores were calculated using Canadian data [17].

#### Demographics

Age, gender, ethnicity, household income, and maternal education data were collected from parents at baseline using adapted questions from the Canadian Community Health Survey [33]. Parents were asked to select their cultural and racial background from a list of 13 ethnic categories. Responses were re-categorized to: 1) White; 2) Chinese/South East Asian; 3) South Asian; 4) Aboriginal; and 5) other. Maternal educational and household income were grouped into categories as displayed in Table 1.

**Table 1:** Socio-demographic characteristics of participants (*n* = 159)

	Adolescent	Parent^a^
Mean (SD) age	13.2 (1.8)	45.8 (6.2)
Gender [n (%)]		
Male	68 (42.8)	24 (15.1)
Female	91 (57.2)	135 (84.9)
Mean (SD) baseline BMI (kg/m^2^)	30.7 (5.9)	30.0 (7.2)
Mean (SD) baseline BMI z-score	2.67 (0.82)	
Mean (SD) baseline waist circumference (cm)	93.3 (13.8)	92.1 (16.3)
Mean (SD) baseline waist circumference z-score	4.37 (2.05)	
Parent ethnicity [n (%)]		
White		75 (48.1)
East/Southeast Asian		23 (14.7)
South Asian		20 (12.8)
Aboriginal		13 (8.3)
Other		25 (16.0)
Household income [n (%)]		
≤ $40,000		29 (18.8)
$40,001–$80,000		47 (30.5)
$80,001–$120,000		44 (28.6)
$120,001+		34 (22.1)
Maternal education [n (%)]		
High school or less		30 (19.1)
Trade certificate/diploma		57 (36.3)
Bachelor’s degree		33 (21.0)
Above bachelor’s		37 (23.6)

^a^Numbers may not add up to total N due to missing data

### Analysis

Linear mixed models were conducted to assess the effect of adolescent participation and self-monitoring rates on change in adolescent BMI and WC z-scores over time. Adolescent participation rate and self-monitoring were analyzed separately for the two outcome variables. Included in each model were time (as a random effect), an interaction between time and the main independent variable (participation rate or self-monitoring) controlling for all baseline socio-demographic variables (i.e., adolescent age and gender and household income, maternal education, and ethnicity). Linear mixed models allows for the inclusion of all available data regardless of the amount of missing data. Analyses of the data using multiple imputation techniques found similar results and therefore are not reported. The moderating effect of parent participation was examined by including all three-way and two-way interaction terms between parent participation, adolescent participation and time. Multivariable regression analyses were conducted to assess predictors of adolescent adherence and self-monitoring rates using all socio-demographic variables and parental participation and self-monitoring. All analyses were conducted using Stata v.13.1.

## Results

Sample characteristics are displayed in Table 1. Of the adolescents, 81% were obese. In contrast, 34% of parents were overweight and 41% were obese. Of the 33 weeks that adolescents and parents were asked to login to their respective websites, adolescents logged into the website an average of 13.4 weeks, and parents logged into the website an average of 7.5 weeks (Table 2).

**Table 2:** Study participation statistics for adolescents and parents

	Mean (SD)	Median (IQR)^a^
Adolescent		
Number of weeks logged into website	13.4 (11.4)	11 (3–24)
Percent of weeks logged into website	40.5 (34.6)	33.3 (9.1–72.7)
Number of weeks self-monitoring was entered	8.3 (9.9)	4 (0–13)
Percent of weeks self-monitoring was entered	24.3 (29.2)	11.8 (0–38.2)
Participation Rate		
Baseline to 4 months	38.1 (32.3)	33.2 (7.1–68.2)
4 months to 8 months	18.0 (24.2)^b^	4.7 (0–35.0)
Baseline to 8 months	28.4 (26.8)	20.5 (4.8–46.5)
Self-monitoring Rate		
Baseline to 4 months	36.3 (36.2)	23.5 (0–70.6)
4 months to 8 months	12.4 (27.3)^b^	0 (0–5.9)
Baseline to 8 months	24.3 (29.2)	11.8 (0–38.2)
Parent		
Number of weeks logged into website	7.5 (9.7)	3 (0–12)
Percent of weeks logged into website	22.3 (29.5)	9.1 (0–36.4)
Number of weeks self-monitoring was entered	4.5 (8.3)	0 (0–5)
Percent of weeks self-monitoring was entered	13.2 (24.5)	0 (0–14.7)
Participation Rate		
Baseline to 4 months	30.9 (34.7)	17.6 (0–58.8)
4 months to 8 months	14.2 (27.8)^b^	0 (0–6.3)
Baseline to 8 months	22.8 (29.5)	9.1 (0–36.4)
Self-monitoring Rate		
Baseline to 4 months	18.5 (29.3)	0 (0–23.5)
4 months to 8 months	8.0 (23.1)^b^	0 (0–0)
Baseline to 8 months	13.2 (24.5)	0 (0–14.7)

^a^Inter-quartile range

^b^Significantly different compared to baseline to 4 months

Table 2 displays the mean and median participation and self-monitoring rates of adolescents and parents. On average, adolescents and parents completed 28% (proportion of web-pages viewed) and 23% (proportion of weeks logged in) of the intervention, respectively. The participation rate was significantly higher during the first 4 months than the last 4 months for both adolescents (38% vs. 18%) and parents (31% vs. 14%). Fifteen (9.4%) adolescents and 50 parents (31.5%) did not login to the intervention website during the entire study period. On average, adolescents and parents entered self-monitoring data on at least one day for at least one behaviour for 24% and 13% of the weeks during the study period, respectively. Forty-one adolescents (26%) and 81 parents (51%) did not enter any self-monitoring information during the entire study period.

In multivariable regression analyses, parent adherence rate was significantly associated with adolescent participation rate such that a 10% increase in parent participation rate was associated with a 6.1% increase in adolescent participation rate (Table 3). Similarly, parent self-monitoring was significantly associated with adolescent self-monitoring such that a 10% increase in parent self-monitoring was associated with a 5.4% increase in adolescents self-monitoring.

**Table 3:** Predictors of adolescent participation and self-monitoring rates^a^

	Adherence			Self-monitoring		
	b	SE	β	b	SE	β
Parent participation	0.606**	0.069	0.662	0.016	0.085	0.016
Parent self-monitoring	−0.038	0.081	−0.035	0.535**	0.101	0.450

^a^Models adjusted for baseline adolescent age, gender, BMI z-score, and waist circumference z-score, parental ethnicity, maternal education, and household income

*p < 0.05; **p < 0.01

**Fig. 1:**
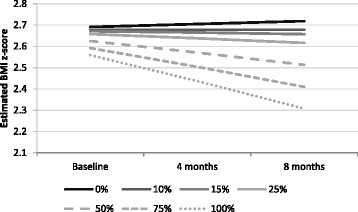
Trajectories of BMI z-score over the study period by participation rate

Table 4 displays the results of the linear mixed models predicting change in BMI z-score and WC z-score. A significant time-by-participation rate interaction (*p* < 0.01) effect was found on BMI z-score such that adolescents with high levels of participation had a decreasing trajectory of BMI z-score and those with low levels of participation had a stable or increasing trajectory of BMI z-score. Differences in BMI z-score trajectory by participation rate can be found in Fig. 1. A decreasing trajectory started at participation rates greater than 10%. No other significant interaction effect was found among the other models. The inclusion of parent participation as an interaction term with adolescent participation and time was not significant and was therefore left out of the model.

**Table 4:** Results from linear mixed-models predicting BMI z-score and waist circumference z-score^a^

	BMI z-score		Waist circumference z-score	
	Participation	Self-monitoring	Participation	Self-monitoring
Fixed effects				
Intercept	3.381 (0.536)**	3.400 (0.533)**	4.041 (1.291)**	4.153 (1.286)**
Time (weeks)	0.001 (0.001)	−0.002 (0.001)	0.000 (0.008)	−0.010 (0.007)
Participation rate (%)	−0.124 (0.231)		−0.212 (0.578)	
Time x Participation rate	−0.008 (0.003)**		−0.018 (0.017)	
Self-monitoring rate (%)		−0.290 (0.210)		−0.711 (0.525)
Time x Self-monitoring rate		−0.002 (0.003)		0.009 (0.014)
Random effects				
Intercept standard deviation	0.721 (0.042)	0.718 (0.042)	1.631 (0.123)	1.618 (0.122)
Slope standard deviation	0.007 (0.001)	0.007 (0.001)	0.027 (0.007)	0.027 (0.007)
Residual standard deviation	0.116 (0.008)	0.116 (0.008)	0.868 (0.063)	0.869 (0.064)

^a^Table displays coefficients and standard errors (in parentheses); Models adjusted for baseline adolescent age, adolescent gender, household income, maternal education, and ethnicity

*p < 0.05; **p < 0.01

## Discussion

This study found that adolescents who had greater participation in the e-health lifestyle intervention had a greater decrease in their BMI z-score trajectory at 8-months than those who participated less. Specifically, adolescents who viewed more than 10% of the intervention materials stabilized their BMI z-score trajectories but those who viewed more content saw a greater decrease in their BMI z-score trajectories – e.g., viewing 50% of the content resulted in an average BMI z-score reduction of 0.1 at 8-months. This represents the first web-based intervention study among adolescents that documented a change in BMI z-score as previous studies found attendance or utilization of program components resulted in change in health behaviours (PA and nutrition outcomes) without a change in BMI z-score [15, 28, 43]. The short duration of previous e-Health interventions may explain why weight change was not observed in past studies [15]. In addition, parent participation did not appear to moderate the effect of adolescent participation on weight outcomes. Finally, counter to what others have found, [12, 21, 25, 28] utilization of the self-monitoring tools by either parents or adolescents was not related to adolescents’ change in BMI or WC z-scores. It may be that the self-monitoring tools utilized in this intervention were not as engaging as those included in other studies as a high percentage of adolescents and parents did not use them (26% and 51%, respectively).

Contrary to expectations, parental participation did not influence the outcomes of the intervention as it did not moderate adolescents change in BMI z-score even though parental participation was associated with adolescents’ participation. This finding contradicts what others have found as family-based intervention have improved the success of traditional clinical interventions [22, 26]. However, these interventions focused on pre-adolescents or younger children, targeted the parents, and were delivered in person and in group-based settings and ensured that parents were actively engaged in the intervention [3]. One possible mechanisms through which parental participation could have influenced the outcomes of the current intervention is by changing the home environment to better support their adolescent’s health behaviors, [11] as the parent site provided them with advice on how to support the challenges and goals their adolescents were expected to achieve that week. While this e-health intervention targeted both the adolescents and their parents, the e-health context may be less successful at engaging and promoting supportive interactions between adolescents and their parents. While parents can influence their adolescents’ participation in the intervention, the approach they use to achieve this may not result in the desired effect as observed in this study. For example, if parents are pressuring their adolescents to participate it can explain why parental participation did not influence the outcomes of the intervention as controlling strategies have been found to be less effective than autonomy supportive approaches in achieving desired health behaviours [7]. For example, a number of observational and longitudinal studies have shown that parents who use controlling practices such as pressuring the child to eat healthier food result in poor self-regulatory behaviours such as eating in the absence of hunger [8, 9]. Previous studies emphasize the importance of supporting change in children’s health behaviours at the household level; [3, 22] however, this study suggests the need in helping parents use more autonomy supportive parenting practices as it may explain why their engagement did not positively influence the outcome of the intervention. Future e-health interventions should better support parent/adolescent interactions, such as providing parents with the skills to use autonomy supportive approaches as a way to better support their adolescent and ultimately improve the efficacy of these interventions.

As internet access in households is becoming increasingly common (in 2012 at least 83% of Canadian and U.S., households had internet at home), [34] online delivery of interventions has the potential to broadly improve health outcomes among a wider segment of the population [2, 4, 6]. However, participation rates for online interventions have been quite variable [18]. Overall, participation in the present e-health intervention was sub-optimal for both adolescents and parents and appears much lower than other web-based interventions that report a 50% participation rate on average [18]. However, it is somewhat difficult to compare participation rates across studies as most use login rates versus percent of content viewed which is what was reported for the adolescents in the current study. The login rate for this study, defined as the number of weekly logins over the total number of weeks of the study, was 40.5% for the adolescents and 22.3% for the parents. In addition, participation rates are likely higher in shorter interventions than in longer interventions. In fact, this study found much higher participation rates in the first four months of the intervention than in the last four months. Nonetheless, strategies to improve participation of adolescents may benefit e-health interventions.

Utilization of the self-monitoring tools that were part of the e-health intervention was not found to have an effect on adolescents BMI z-score trajectory, even though there is evidence that such strategy is important to include in interventions [12, 21, 25, 28]. This study documented utilization of the self-monitoring tools by determining whether the participants entered information into the e-health program; however, both parents and adolescents could have used alternative ways of self-monitoring which was not captured by the e-health program (such as using the technique without actually entering the information in the program). This e-health program had different tracking forms for each behavior (steps, screen time, and dietary behaviours) and participants were not expected to use them every week. Perhaps using a single tracking form and setting the expectation that it be used every week would have had a different impact on the outcomes and can partly explain the findings and discrepancies with others studies [12, 21, 25, 28].

There are several limitations to this study. First, the sample included families with overweight and obese adolescents that volunteered to participate in the intervention limiting the generalizability to this population which is typical of similar studies. Second, adolescent’s participation measured whether they viewed the content but it could not be determined whether they fully read the content. Third, this study did not include a control group; therefore, the effect of the intervention on change in BMI z-score must be taken with caution. However, participants that had higher participation rates showed a greater reduction in BMI z-score indicating a potential effect from the intervention. Lastly, the study did not take into account self-perceived weight status which has been shown to be associated with intention to prevent weight gain [40].

## Conclusions

In conclusion, this study found a dose-response relationship between adolescent participation to an e-health lifestyle intervention with BMI trajectory – finding a greater decrease in BMI z-score among overweight or obese adolescents with increased participation. Parental participation influenced their adolescents’ participation; however, it did not influence their adolescents’ reduction in BMI. Future interventions could benefit from exploring potential mechanisms to improve adherence of adolescents in e-health interventions. With improved adherence, these results suggest that e-health lifestyle interventions can be an effective strategy to beneficially alter the weight trajectory of overweight and obese adolescents. In addition, this study highlights the need to develop strategies that promote both active and supportive engagement of parents in e-health interventions as a way to further increase the efficacy of these interventions.

## Abbreviations

BC: British Columbia
BMI: Body mass index
PA: Physical activity
SD: Standard deviation
WC: Waist circumference
WHO: World Health Organization
